# Comparison of the Gait Biomechanical Constraints in Three Different Type of Neuromotor Damages

**DOI:** 10.3389/fnhum.2022.822205

**Published:** 2022-03-29

**Authors:** Silvia Minosse, Martina Favetta, Alberto Romano, Alessandra Pisano, Susanna Summa, Tommaso Schirinzi, Gessica Vasco, Enrico Castelli, Maurizio Petrarca

**Affiliations:** ^1^Department of Neurorehabilitation and Robotics, Movement Analysis and Robotics Laboratory (MARLab), “Bambino Gesù” Children’s Hospital, IRCCS, Rome, Italy; ^2^Department of Biomedicine and Prevention, University of Rome “Tor Vergata”, Rome, Italy; ^3^Department of Systems Medicine, University of Rome “Tor Vergata”, Rome, Italy

**Keywords:** gait, cerebral palsy, Charcot-Marie-Tooth, Duchenne Muscular Dystrophy, absolute angle, biomechanical constraints

## Abstract

**Background and Objective:**

Absolute angle represents the inclination of a body segment relative to a fixed reference in space. This work compares the absolute and relative angles for exploring biomechanical gait constraints.

**Methods:**

Gait patterns of different neuromotor conditions were analyzed using 3D gait analysis: normal gait (healthy, H), Cerebral Palsy (CP), Charcot Marie Tooth (CMT) and Duchenne Muscular Dystrophy (DMD), representing central and peripheral nervous system and muscular disorders, respectively. Forty-two children underwent gait analysis: 10 children affected by CP, 10 children by CMT, 10 children by DMD and 12 healthy children. The kinematic and kinetic parameters were collected to describe the biomechanical pattern of participants’ lower limbs. The absolute angles of thigh, leg and foot were calculated using the trigonometric relationship of the tangent. For each absolute series, the mean, range, maximum, minimum and initial contact were calculated. Kinematic and kinetic gait data were studied, and the results were compared with the literature.

**Results:**

Statistical analysis of the absolute angles showed how, at the local level, the single segments (thigh, leg and foot) behave differently depending on the pathology. However, if the lower limb is studied globally (sum of the kinematics of the three segments: thigh, leg and foot), a biomechanical constraint emerges.

**Conclusion:**

Each segment compensates separately for the disease deficit so as to maintain a global biomechanical invariance. Using a model of inter-joint co-variation could improve the interpretation of the clinical gait pattern.

## Introduction

The introduction of gait analysis has enhanced the concept that motor behavior emerges from the body-environment coupling (Kuniyoshi and Suzuki, 2004; Abu-Faraj et al., 2015). Gait analysis is a multi-factorial analysis assessing kinematic, kinetic and electromyographic activities (EMG). These techniques are used to identify the kinematic determinants of human locomotion. Attention was focused on the relationship between the biomechanics of the human body and the brain (Lacquaniti et al., 1999) as these two elements do not work at the same logical level. The body biomechanics represents a given system of constraints, as shown by Collins et al. (2005) with his bipedal modeling of dynamic passive gait. In living creatures, the body is characterized by a redundancy of degree of freedom (DoF), raising the problem about their control during dynamic functional activities (Davies, 1968). The Central Nervous System fine-tunes the synergic actions of the muscles controlling the redundancy of the body DoF (Davies, 1968). In this perspective, the brain is the “medium” of the relationship between the organism and the environment, as explained by the ecologic approach to motor control and learning (Gibson, 1979). Although this relationship is well established, knowledge concerning the particular strategies used by the brain to mediate between body biomechanics and the environment is limited. The nature of the biomechanical constraints implies that appropriate solutions for walking are limited, and the usual bipedal walking represents a sort of final common path. However, different gait patterns that deviate from the “typical” one are frequently observed in pathologic conditions. It is possible to hypothesize that the way of walking, expressed in pathological conditions, can offer some keys for understanding the invariance rules that bind the biomechanical and neurological mechanisms. As an example, it was demonstrated that patients with hemiplegia and voluntary toe walkers shared the same kinematic, kinetic and EMG patterns (Romkes and Brunner, 2007). To explore the elements of invariance, we decided to analyze the gait pattern of children with different pathologies looking at differences and similarities. We analyzed the gait pattern that emerged from three different types of damage to the neuromotor system caused by Cerebral Palsy (CP), Charcot-Marie-Tooth (CMT) and Duchenne Muscular Dystrophy (DMD). They can be considered paradigmatic examples corresponding to damage involving the upper motor neuron, the lower motor neuron, and the muscles. CP, CMT and DMD children present complex and heterogeneous gait patterns already described using gait analysis tools (Salami et al., 2017; Wojciechowski et al., 2017; Davids et al., 2018; Goudriaan et al., 2018; Romano et al., 2019). To date, there has been no comparative study aimed at defining similarities and differences. The present study was facilitated by the fact that our gait lab is situated within a big clinical research center specialized in following children with rare diseases.

Briefly, CP is characterized by movement disorder, spasticity, muscle weakness, ataxia and rigidity (Armand et al., 2016). It has been reported that the alterations in the selective motor control are the result of failure to control reciprocal activation of the agonist and the antagonist muscles and correlate with the gait inability (Crenna, 1998; Chruscikowski et al., 2017). Their gait alterations are currently classified in literature (Rodda et al., 2004; Cioni et al., 2008) without reaching a consensus.

CMT is a peripheral nervous system disorder. Affected patients show skeletal deformities, distal muscle weakness and atrophy, and sensory impairment leading to walking impairment (Lencioni et al., 2017) which have already been classified in literature (Wojciechowski et al., 2017).

DMD is characterized by a progressive replacement of muscle fibers with fibro-fatty tissue and severe muscular weakness. Progressive muscular degeneration determines the onset of compensatory strategies during walking (Doglio et al., 2011). The gait pattern of this population has been widely described (Sienko Thomas et al., 2010; Goudriaan et al., 2018).

The present study aimed to investigate similarities and differences between the gait patterns of three different pathologic groups of children with CMT, DMD and CP compared to healthy children. The main hypothesis is that the three different gait patterns were influenced by the particular nature of the pathology (central, peripheral and muscular). However, they could also share elements of invariance. This invariance can be induced by body biomechanics and environmental physics, similarly to what identified in studies of lower limb absolute angles in healthy subjects (Borghese et al., 1996; Hamill et al., 2014). Ours is a pilot study in which, besides kinematics, the kinematic gait relative and absolute body segment configurations were compared, searching for differences and similarities, to enhance present knowledge related to the control of bipedal locomotion in pathological conditions.

## Materials and Methods

### Participants

Ten children with CMT, ten children with DMD and ten children with CP were enrolled in the study. The eligibility criteria for this study were: age between 5 and 15 years and independent walking without orthosis. CP is a heterogeneous condition by definition. Consequently, both to reduce CP heterogeneity and to achieve the aims of this study, only participants with Gross Motor Function Classification System I and II were included, with a gait pattern corresponding to group IV of the Rodda et al. (2004) classification (Rodda et al., 2004) and to form IV of the Cioni et al. (2008) gait classification (Cioni et al., 2008). Exclusion criteria were: surgical treatment in the last year and administration of botulin toxin or experimental drug during the previous 6 months.

The group of children with CMT consisted of eight boys and two girls, with an average age of 12.0 (range: 7.0–15.0 years), an average weight of 46.3 (range: 25.0–79.5 kg), an average height of 1.44 (range:1.19–1.69 m) and an average leg length of 0.76 (range: 0.64–0.96 m).

The group of children with DMD consisted of 10 boys, with an average age of 7.7 (range: 5.0–11.0 years), average weight of 27.9 (range: 17.8–46.0 kg), average height of 1.23 (range: 1.04–1.63 m) and average leg length of 0.61 (range: 0.53–0.71 m).

The group of children with CP consisted of five boys and five girls, with an average age of 9.3 (range: 5.6–15.7 years), an average weight of 29.0 (range: 18.5–49.5 kg), an average height of 1.28 (range: 0.96–1.57 m) and average leg length of 0.69 (range: 0.48–0.87 m).

Reference data were collected in a group of twelve children, seven boys and five girls, without any neurological or neuromuscular problems, with an average age of 10.9 (range: 6.0–14.5), an average weight of 39.2 (range: 22.0–60.3 kg), average height 1.43 (range: 1.15–1.68 m) and average leg length 0.79 (range: 0.63–0.94 m).

All the children and their parents gave informed consent before starting the evaluation sessions. The Ethics Committee of the Hospital authorized the study. For an exhaustive clinical description of the pathologic groups see Table 1.

**TABLE 1:** Clinical scale and six Minute Walking Test (6MWT): For Cerebral Palsy (CP) the clinical scale used is the Gross Motor Function Measure-66 (GMFM-66), for Charcot Marie Tooth (CMT) the clinical scale is the CMT Pediatric Scale (CMTPedS), and for Duchenne Muscular Dystrophy (DMD) the clinical scale is the NorthStar Ambulatory Assessment (NSAA).

**CP**	**GMFM-66 (%)**	**6MWT (m)**
CP1	51.85	280
CP2	78.28	335
CP3	98	501
CP4	66.69	352
CP5	70.39	375
CP6	96	443
CP7	86.52	331
CP8	75.34	390
CP9	100	414
CP10	71.22	345
CP11	86.52	395
**CMT**	**CMTPedS**	
CMT1	21	555
CMT2	18	562
CMT3	16	606
CMT4	25	550
CMT5	13	462
CMT6	18	512
CMT7	28	440
CMT8	31	375
CMT9	22	516
CMT10	28	464
**DMD**	**NorthStar Ambulatory Assessment**	
DMD1	31	564
DMD2	-	385
DMD3	28	335
DMD4	23	406
DMD5	26	364
DMD6	12	340
DMD7	31	425
DMD8	15	386
DMD9	31	464
DMD10	31	550

### Gait Analysis

Gait analysis was performed using an eight-camera motion capture system (Vicon MX, United Kingdom) with sampling rates of 200 Hz and two force plates (AMTI, Or6-6, United States) with sampling rates of 1 kHz. The two force plates were situated in the middle portion of a 10 m walkway. Plug-in-Gait protocol for reconstructing a body kinematic and kinetic model was used. Participants walked barefoot at their self-selected speed. For each child, three representative gait cycles were considered. Kinematic and kinetic temporal series were normalized to the stride duration. Kinetic data were normalized to the subject’s weight. In addition, we evaluated spatio-temporal parameters, walking velocity and step length were normalized to leg length.

**FIGURE 1:**
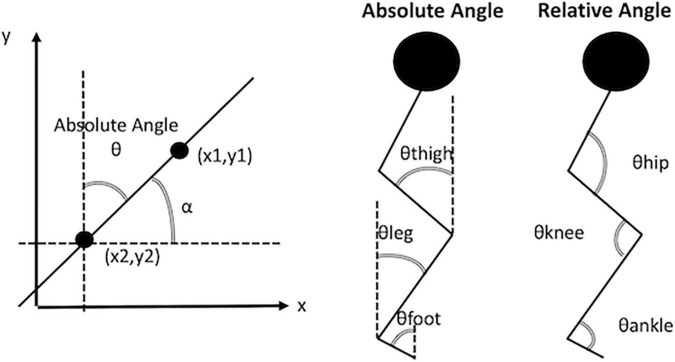
Illustrative example of absolute angles and relative angles. In the panel (right) the absolute angle ⊖ is the complementary angle of angle α. The angle α is obtained from the arctangent of the slope of the line passing through the points (x1, y1) and (x2, y2). In the panel (center) are depicted the absolute angles of thigh, leg, and foot. In the panel (left) are depicted the relative angle of the hip, the knee and the ankle.

The lower limb absolute angles (segment angles) that describe the segment’s orientation in space were also calculated. Absolute angles are computed using the trigonometric relationship of the tangent. The tangent is equal to the angular coefficient obtained with a linear fit between the proximal endpoints of the segment. Schematic illustrations of the absolute angles (thigh, leg and foot) and relative angles (hip, knee and ankle) are reported in Figure 1. The following list of variables was selected from absolute angle curves of thigh, leg and foot: initial contact, average, range, maximum and minimum. The sum of the absolute angles (thigh, leg and foot) was then evaluated to determine the biomechanical constraints in lower limbs. The initial contact, average, range, maximum and minimum were calculated from the total absolute angle. See Tables 2, 3 for a detailed report of the kinematic and kinetic parameters.

**TABLE 2:** The list of the kinematic and kinetic variables of the lower limb (pelvis, hip, knee and ankle) and the absolute angles of thigh, leg and foot.

**Foot progression angle**	
$F\,P\,A_{I\,C}$	Foot progression angle at initial contact
$F\,P\,A_{a}$	Average of foot progression angle
$F\,P\,A_{r}$	Range of foot progression angle
$F\,P\,A_{m\,a\,x}$	Maximum rotation of foot progression angle
$F\,P\,A_{m\,i\,n}$	Minimum rotation of foot progression angle
$F\,P\,A_{max.\%}$	% of Gait cycle corresponding of maximum rotation of foot progression angle
$F\,P\,A_{min.\%}$	% of Gait cycle corresponding of minimum rotation of foot progression angle
**Pelvis**	
$P_{\mathrm{IC}}^{T}$	Pelvic tilt angle at initial contact
$P_{a}^{T}$	Average pelvic tilt angle
$P_{r}^{T}$	Range pelvic tilt angle
$P_{\mathrm{IC}}^{O}$	Pelvic obliquity angle at initial contact
$P_{a}^{O}$	Average pelvic obliquity angle
$P_{r}^{O}$	Range pelvic obliquity angle
**Hip**	
$H_{I\,C}^{F/E}$	Hip flexion/extension angle at initial contact
$H_{a}^{F/E}$	Average hip flexion/extension angle
$H_{r}^{F/E}$	Range hip flexion/extension angle
$H_{\max}^{F}$	Maximum hip flexion angle
$H_{\max}^{E}$	Maximum hip extension angle
$H_{\max.\%}^{F}$	% of Gait cycle corresponding of maximum hip flexion angle
$H_{\max.\%}^{E}$	% of Gait cycle corresponding of maximum hip extension angle
$H_{I\,C}^{A\,d/A\,b}$	Hip adduction/abduction angle at initial contact
$H_{a}^{A\,d/A\,b}$	Average hip adduction/abduction angle
$H_{r}^{A\,d/A\,b}$	Range hip adduction/abduction angle
$H_{m\,a\,x}^{A\,d}$	Maximum hip adduction angle
$H_{m\,a\,x}^{A\,b}$	Maximum hip abduction angle
$H_{max.\%}^{A\,d}$	% of Gait cycle corresponding of maximum hip adduction angle
$H_{max.\%}^{A\,b}$	% of Gait cycle corresponding of maximum hip abduction angle
$H\,P_{m\,a\,x}^{G.s\,t}$	Maximum hip power generation during stance phase
$H\,P_{m\,a\,x}^{A.s\,t}$	Maximum hip power absorbed during stance phase
$H\,P_{m\,a\,x}^{G.s\,w}$	Maximum hip power generation during swing phase
**Knee**	
$K_{I\,C}^{F/E}$	Knee flexion/extension angle at initial contact
$K_{a}^{F/E}$	Average knee flexion/extension angle
$K_{r}^{F/E}$	Range knee flexion/extension angle
$K_{m\,a\,x}^{F.s\,t}$	Maximum knee flexion angle during stance phase
$K_{m\,a\,x}^{E.s\,t}$	Maximum knee extension angle during stance phase
$K_{m\,a\,x}^{F.s\,w}$	Maximum knee flexion angle during swing phase
$K_{max.\%}^{F.s\,t}$	% of Gait cycle corresponding of maximum knee flexion angle during stance phase
$K_{max.\%}^{E.s\,t}$	% of Gait cycle corresponding of maximum knee extension angle during stance phase
$K_{max.\%}^{F.s\,w}$	% of Gait cycle corresponding of maximum knee flexion angle during swing phase
$K\,P_{m\,a\,x}^{G.s\,t}$	Maximum knee power generation during stance phase
$K\,P_{m\,a\,x}^{A.s\,t}$	Maximum knee power absorbed during stance phase
$K\,P_{m\,a\,x}^{G.s\,w}$	Maximum knee power generation during swing phase
**Ankle**	
$A_{I\,C}^{D/P}$	Ankle dorsal/plantar angle at initial contact
$A_{a}^{D/P}$	Average ankle dorsal/plantar angle
$A_{r}^{D/P}$	Range ankle dorsal/plantar angle
**Ankle**	
$A_{m\,a\,x}^{D.s\,t}$	Maximum ankle dorsal angle during stance phase
$A_{m\,a\,x}^{P.s\,t}$	Maximum ankle plantar angle during stance phase
$A_{m\,a\,x}^{D.s\,w}$	Maximum ankle dorsal angle during swing phase
$A_{max.\%}^{D.s\,t}$	% of Gait cycle corresponding of maximum ankle dorsal angle during stance phase
$A_{max.\%}^{P.s\,t}$	% of Gait cycle corresponding of maximum ankle plantar angle during stance phase
$A_{max.\%}^{D.s\,w}$	% of Gait cycle corresponding of maximum ankle dorsal angle during swing phase
$A\,P_{m\,a\,x}^{G.s\,t}$	Maximum ankle power generation during stance phase
$A\,P_{m\,a\,x}^{A.s\,t}$	Maximum ankle power absorbed during stance phase
**Absolute angles**	
**Thigh**	
*T*_*IC*_	Absolute thigh angle at initial contact
*T_a_*	Average of absolute thigh angle
*T_r_*	Range of absolute thigh angle
*T*_*max*_	Maximum of absolute thigh angle
*T*_*min*_	Minimum of absolute thigh angle
**Leg**	
*L*_*IC*_	Absolute leg angle at initial contact
*L_a_*	Average of absolute leg angle
*L_r_*	Range of absolute leg angle
*L*_*max*_	Maximum of absolute leg angle
*L*_*min*_	Minimum of absolute leg angle
**Foot**	
*F*_*IC*_	Absolute foot angle at initial contact
*F_a_*	Average of absolute foot angle
*F_r_*	Range of absolute foot angle
*F*_*max*_	Maximum of absolute foot angle
*F*_*min*_	Minimum of absolute foot angle

**TABLE 3:** Differences between the median values of two different pathologies: Healthy (H), Cerebral Palsy (CP), Charcot Marie Tooth (CMT) and Duchenne Muscular Dystrophy (DMD).

**Variables**	**H–CMT**		**H–DMD**		**H–CP**		**CMT–DMD**		**CMT–CP**		**DMD–CP**	
	**Δ**	**p**	**Δ**	**p**	**Δ**	**p**	**Δ**	**p**	**Δ**	**p**	**Δ**	**p**
Foot Off (%)	−0.14	ns	0.42	ns	−1.80	ns	0.56	ns	−1.66	ns	−2.22	ns
Stride Velocity (1/s)	0.17	ns	−0.19	ns	0.15	ns	−**0.36**	**0.042**	−0.03	ns	**0.34**	**0.015**
Stride Length (-)	0.07	ns	0.00	ns	0.15	Ns	−0.08	ns	0.08	ns	0.16	ns
Stride Time (s)	−0.10	ns	0.11	ns	−0.02	ns	**0.21**	**0.002**	0.08	ns	−**0.13**	**0.036**
Stride Width (-)	−0.01	ns	−**0.06**	**0.017**	−**0.12**	**0.001**	−0.05	ns	−0.11	ns	−0.06	ns
$F\,P\,A_{I\,C}$ (deg)	3.22	ns	−2.73	ns	−11.90	ns	−5.95	ns	−**15.12**	**0.002**	−9.17	ns
$F\,P\,A_{a}$ (deg)	2.67	ns	−3.74	ns	−13.67	ns	−6.40	ns	−**16.34**	**0.002**	−9.93	ns
$F\,P\,A_{r}$ (deg)	−1.48	ns	−0.70	ns	−**11.19**	**0.002**	0.78	ns	−**9.71**	**0.024**	−**10.49**	**0.042**
$F\,P\,A_{m\,a\,x}$ (deg)	2.06	ns	−4.07	ns	−**16.84**	**0.048**	−6.12	ns	−**18.90**	**0.004**	−12.77	ns
$F\,P\,A_{m\,i\,n}$ (deg)	1.88	ns	−3.11	ns	−5.22	ns	−4.99	ns	−**7.11**	**0.040**	−2.12	ns
$F\,P\,A_{max.\%}$ (%)	−5.08	ns	10.08	ns	-0.40	ns	15.16	ns	4.68	ns	−10.48	ns
$F\,P\,A_{min.\%}$ (%)	0.95	ns	0.78	ns	−4.48	ns	−0.17	ns	−5.43	ns	−5.26	ns
$P_{\mathrm{IC}}^{T}$ (deg)	1.60	ns	−4.33	ns	−3.92	ns	−5.92	ns	−5.51	ns	0.41	ns
$P_{a}^{T}$ (deg)	1.55	ns	−4.09	ns	−**7.20**	**0.007**	−5.64	ns	−**8.75**	**0.005**	−3.11	ns
$P_{r}^{T}$ (deg)	0.22	ns	−1.35	ns	−**4.63**	**<0.001**	−1.57	ns	−**4.86**	**0.008**	−3.29	ns
$P_{\mathrm{IC}}^{O}$ (deg)	−0.49	ns	−1.75	ns	2.08	ns	−1.25	ns	2.58	ns	**3.83**	** < 0.001**
$P_{a}^{O}$ (deg)	0.04	ns	−0.01	ns	0.15	ns	−0.05	ns	0.11	ns	0.16	ns
$P_{r}^{O}$ (deg)	2.60	ns	−2.18	ns	−2.56	ns	−**4.78**	**0.027**	−**5.16**	**0.018**	−0.38	ns
$H_{I\,C}^{F/E}$(deg)	8.74	ns	−1.96	ns	−10.85	ns	−10.70	ns	−**19.59**	** < 0.001**	−8.89	ns
$H_{a}^{F/E}$(deg)	**7.59**	**0.049**	−1.92	ns	−7.36	ns	−9.50	ns	−**14.95**	** < 0.001**	−5.45	ns
$H_{r}^{F/E}$(deg)	−0.62	ns	−0.82	ns	−5.62	ns	−0.20	ns	−5.01	ns	−4.80	ns
$H_{\max}^{F}$(deg)	10.19	ns	−0.94	ns	−**12.61**	**0.014**	−11.14	ns	−**22.80**	** < 0.001**	−11.66	ns
$H_{\max}^{E}$(deg)	**9.28**	**0.018**	1.01	ns	−5.76	ns	−8.27	ns	−**15.03**	** < 0.001**	−6.76	ns
$H_{\max.\%}^{F}$(%)	8.73	ns	−0.29	ns	−2.20	ns	−9.02	ns	−10.93	ns	−1.91	ns
$H_{\max.\%}^{E}$(%)	−0.96	ns	−0.20	ns	1.64	ns	0.76	ns	2.60	ns	1.84	ns
$H_{I\,C}^{A\,d/A\,b}$(deg)	−2.28	ns	−1.64	ns	2.66	ns	0.64	ns	**4.94**	**0.001**	**4.30**	**0.002**
$H_{a}^{A\,d/A\,b}$(deg)	−0.35	ns	1.08	ns	1.71	ns	1.44	ns	2.06	ns	0.63	ns
$H_{r}^{A\,d/A\,b}$(deg)	3.07	ns	−2.65	ns	1.74	ns	−**5.72**	** < 0.001**	−1.33	ns	**4.40**	**0.030**
$H_{m\,a\,x}^{A\,d}$(deg)	1.66	ns	−2.21	ns	3.00	ns	−3.86	ns	1.35	ns	5.21	ns
$H_{m\,a\,x}^{A\,b}$(deg)	−1.04	ns	3.30	ns	2.13	ns	**4.34**	**0.049**	3.17	ns	−1.16	ns
$H_{max.\%}^{A\,d}$(%)	−**18.43**	**0.005**	−6.43	ns	−**12.92**	**0.012**	12.00	ns	5.51	ns	−6.49	ns
$H_{max.\%}^{A\,b}$(%)	0.57	ns	0.12	ns	3.64	ns	−0.44	ns	3.07	ns	3.51	ns
$H\,P_{m\,a\,x}^{G.s\,t}$(W/kg)	0.23	ns	0.20	ns	−0.39	ns	−0.04	ns	−**0.62**	**0.013**	−**0.58**	**0.002**
$H\,P_{m\,a\,x}^{A.s\,t}$(W/kg)	0.13	ns	−0.06	ns	0.09	ns	−0.20	ns	−0.04	ns	0.15	ns
$H\,P_{m\,a\,x}^{G.s\,w}$ (W/kg)	0.48	ns	0.34	ns	0.07	ns	−0.14	ns	−0.41	ns	−0.27	ns
$K_{I\,C}^{F/E}$(deg)	**10.80**	**0.019**	6.22	ns	−19.68	ns	−4.58	ns	−**30.48**	** < 0.001**	−**25.91**	** < 0.001**
$K_{a}^{F/E}$(deg)	**14.01**	**<0.001**	5.92	ns	−3.43	ns	−8.09	ns	−**17.44**	**<0.001**	−9.35	ns
$K_{r}^{F/E}$(deg)	1.71	ns	−3.41	ns	3.84	ns	−5.13	ns	2.12	ns	7.25	ns
$K_{m\,a\,x}^{F.s\,t}$(deg)	**11.29**	**0.004**	7.65	ns	−14.99	ns	−3.64	ns	−**26.29**	** < 0.001**	−**22.65**	**0.004**
$K_{m\,a\,x}^{E.s\,t}$(deg)	**14.13**	** < 0.001**	7.61	ns	3.60	ns	−6.52	ns	−**10.54**	**0.004**	−4.01	ns
$K_{m\,a\,x}^{F.s\,w}$(deg)	**14.94**	** < 0.001**	2.57	ns	2.71	Ns	−**12.37**	**0.024**	−**12.24**	**0.019**	0.14	ns
$K_{max.\%}^{F.s\,t}$(%)	1.71	ns	−0.33	ns	7.47	< 0.001	−2.04	ns	**5.76**	**0.042**	**7.80**	**0.009**
$K_{max.\%}^{E.s\,t}$(%)	−7.76	ns	1.86	ns	3.40	ns	9.62	ns	11.16	ns	1.54	ns
$K_{max.\%}^{F.s\,w}$(%)	−0.85	ns	−0.69	ns	−**7.83**	** < 0.001**	0.15	ns	−**6.98**	**0.019**	−**7.14**	** < 0.001**
$K\,P_{m\,a\,x}^{G.s\,t}$(W/kg)	−0.36	ns	0.03	ns	−0.53	ns	0.39	ns	−0.17	ns	−0.56	ns
$K\,P_{m\,a\,x}^{A.s\,t}$(W/kg)	−0.41	ns	−0.33	ns	0.64	ns	0.08	ns	**1.05**	**0.006**	**0.97**	**0.010**
$K\,P_{m\,a\,x}^{G.s\,w}$(W/kg)	0.05	ns	0.13	ns	0.16	ns	0.08	ns	0.11	ns	0.03	ns
$A_{I\,C}^{D/P}$(deg)	**6.76**	**0.006**	5.58	ns	**8.26**	**0.003**	−1.18	ns	1.50	ns	2.67	ns
$A_{a}^{D/P}$(deg)	**5.66**	**0.029**	5.04	ns	**7.62**	**0.009**	−0.62	ns	1.96	ns	2.58	ns
$A_{r}^{D/P}$(deg)	7.03	ns	1.70	ns	−5.25	ns	−5.33	ns	−**12.27**	**0.014**	−6.95	ns
$A_{m\,a\,x}^{D.s\,t}$(deg)	**6.00**	**0.034**	5.85	ns	**9.03**	**0.017**	−0.15	ns	3.04	ns	3.19	ns
$A_{m\,a\,x}^{P.s\,t}$(deg)	1.63	ns	6.78	ns	14.24	ns	5.15	ns	12.62	ns	7.47	ns
$A_{m\,a\,x}^{D.s\,w}$(deg)	**8.58**	** < 0.001**	**5.65**	**0.022**	**7.52**	**0.002**	−2.94	ns	−1.07	ns	1.87	ns
$A_{max.\%}^{D.s\,t}$(%)	−1.98	ns	4.02	ns	13.75	ns	6.00	ns	15.73	ns	9.73	ns
$A_{max.\%}^{P.s\,t}$(%)	1.44	ns	−1.88	ns	−2.78	ns	−3.32	ns	−4.22	ns	−0.90	ns
$A_{max.\%}^{D.s\,w}$(%)	−1.09	ns	−2.75	ns	−2.86	ns	−1.66	ns	−1.77	ns	−0.10	ns
$A\,P_{m\,a\,x}^{G.s\,t}$(W/kg)	0.76	ns	0.75	ns	**1.23**	** < 0.001**	0.00	ns	0.47	ns	0.47	ns
$A\,P_{m\,a\,x}^{A.s\,t}$(W/kg)	0.27	ns	0.06	ns	**0.38**	** < 0.001**	−0.21	ns	0.11	ns	0.32	ns
*T*_*IC*_ (deg)	**7.24**	**0.009**	4.59	ns	−6.95	ns	−2.65	ns	−**14.19**	** < 0.001**	−**11.54**	**0.003**
*T_a_* (deg)	**7.37**	** < 0.001**	**5.06**	**0.016**	−2.68	ns	−2.31	ns	−**10.06**	**0.001**	−**7.74**	**0.039**
*T_r_* (deg)	−0.01	ns	−0.11	ns	−6.60	ns	−0.10	ns	−6.58	ns	−6.48	ns
*T*_*max*_ (deg)	**7.55**	**0.013**	3.97	ns	−8.86	ns	−3.58	ns	−**16.41**	** < 0.001**	−**12.83**	**0.003**
*T*_*min*_ (deg)	**8.38**	**0.001**	**5.42**	**0.038**	−2.29	ns	−2.96	ns	−**10.67**	**0.002**	−7.71	ns
*L*_*IC*_ (deg)	−4.36	ns	−2.47	ns	**11.46**	**0.019**	1.89	ns	**15.82**	** < 0.001**	**13.93**	**0.001**
*L_a_* (deg)	−**5.81**	**0.004**	−2.22	ns	2.59	ns	3.59	ns	**8.40**	** < 0.001**	4.81	ns
*L_r_* (deg)	6.25	ns	1.50	ns	**16.12**	** < 0.001**	−4.75	ns	9.87	ns	**14.62**	**0.002**
*L*_*max*_(deg)	−2.96	ns	−0.61	ns	**11.20**	**0.002**	2.35	ns	**14.16**	** < 0.001**	**11.81**	**0.005**
*L*_*min*_ (deg)	−**8.80**	**0.004**	−3.16	ns	−**4.77**	**0.025**	5.64	ns	4.04	ns	−1.61	ns
*F*_*IC*_ (deg)	−1.75	ns	−3.10	ns	−**10.99**	**0.018**	−1.35	ns	−9.23	ns	−7.88	ns
*F_a_* (deg)	−2.31	ns	3.34	ns	8.26	ns	5.65	ns	10.57	ns	4.92	ns
*F_r_* (deg)	5.57	ns	−5.49	ns	1.42	ns	−11.06	ns	−4.15	ns	6.91	ns
$F_{m\,a\,x}\,\left(\text{deg}\right)$	0.12	ns	0.13	ns	1.31	ns	0.02	ns	1.19	ns	1.17	ns
$F_{m\,i\,n}$(deg)	−6.17	ns	5.57	ns	−0.22	ns	11.73	ns	5.95	ns	−5.78	ns

*Abbreviations as Table 1.*

*Δ, differences between the median values of two different pathologies; *p*, *p*-value corresponding; ns, not significant. Significant values are highlighted in bold characters.*

### Statistics

For each child, we evaluated three kinematic and three kinetic parameters for each representative gait cycle. Finally, we calculated the mean values. The Shapiro–Wilk normality test was used to verify the normal distribution. Since the data were not normally distributed, we assessed a non-parametric statistic. The Wilcoxon signed rank test for paired samples was used to compare the data obtained for the left and right limbs. Since the test was not statistically significant, the average between right and left limbs was calculated for all patients. The Kruskal–Wallis test and a post–hoc with Bonferroni correction determined significant differences in the kinematic (relative and absolute angles) and kinetic parameters between the four groups (CMT, DMD, CP and healthy). A *p* < 0.05 was considered to indicate statistical significance.

## Results

Spatio-temporal, kinematic (relative and absolute angles) and kinetic parameters for the variable illustrated in Table 2 are shown in Table 3.

Children with DMD walked with a statistically significant higher stride velocity and a shorter stride time than those with CMT and CP. Stride width values were significantly larger in DMD and CP children compared to healthy controls. No statistically significant differences in any spatio-temporal parameters were found when comparing the children with CMT with the healthy children or those with CP (Table 3).

**FIGURE 2:**
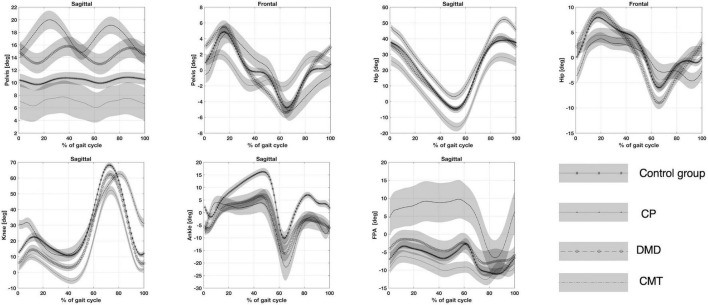
Top panel (from left to right): Pelvis angle on the sagittal and the frontal plane. Hip angle on the sagittal and the frontal plane. Bottom panel (from left to right): Knee angle, Ankle angle on the sagittal plane and Foot progression angle (FPA) on the transversal plane. CP, participants with Cerebral Palsy; CMT, participants with Charcot Marie Tooth; DMD, participants with Duchenne Muscular Dystrophy; normal gait (healthy, H).

The analysis of kinematic pelvic parameters did not show statistically significant differences between healthy controls and children with CMT or DMD (Figure 2). The children with CP presented average and range pelvic tilt angles statistically higher than controls and children with CMT. Although we did not find any statistical significance in this sample, we observed that patients with DMD tended toward a pelvic anteversion and CMT patients toward a pelvic retroversion, as it is shown in the sagittal view of the Pelvis angle in Figure 2 (i.e. the CMT time series angle is the lower curve).

The pelvic obliquity angle at initial contact in children affected by DMD was increased compared to those with CP. The children with CMT showed a reduction in the range of the pelvic obliquity angle compared to DMD and CP groups (Figure 2).

The children with CMT presented the average of the flexion/extension and maximum extension of hip angle lower compared to healthy children (Figure 2). The CP group showed an increase in maximum hip flexion compared to healthy patients. The children affected by CMT showed initial contact, average, and maximum and minimum flexion/extension of the hip angles statistically lower compared to CP children. For the patients affected by CP the initial contact of hip adduction/abduction angle resulted lower and statistically significant when compared to children with DMD and CMT (Figure 2). Patients with DMD showed a range of hip adduction/abduction angle greater than peers with CMT and CP.

The children with CMT showed decreased initial contact, average and maximum and minimum knee flexion angles compared to healthy children (Figure 2). However, the initial contact, average, and maximum knee flexion angles were significantly higher in children with CP than in peers with CMT and DMD.

Ankle dorsal/plantar angle at initial contact, average and maximum ankle dorsal angle during the stance phase were significantly lower in participants with CMT and DMD than in healthy children (Figure 2). The maximum ankle dorsal angle during swing phase values was statistically lower for all pathological groups (CMT, DMD and CP) than for healthy control groups. The ankle dorsal/plantar angle range resulted lower for children with CMT compared to participants with CP.

Increased range and maximum foot progression angles were only observed in children with CP compared with healthy children (Figure 2).

The children with CP presented initial contact, average, range, and maximum and minimum rotation of the foot progression angles significantly higher than children with CMT (Figure 2). We observed a reduction of the range of the foot progression angle for children with DMD compared to children with CP.

**FIGURE 3:**
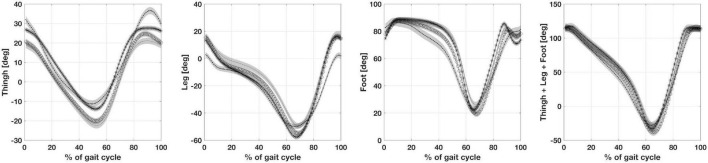
Illustrates from left to right, the absolute angles of thigh, leg, foot and the sum of thigh, leg and foot. CP, participants with Cerebral Palsy; CMT, participants with Charcot Marie Tooth; DMD, participants with Duchenne Muscular Dystrophy; normal gait (healthy, H).

The absolute angle for thigh, leg and foot are shown in Figure 3. There are no statistically significant differences between CMT and DMD groups for absolute angle of thigh, leg and foot. However, there were differences between the children with CMT and peers in the CP and healthy groups for the thigh and leg segments but not for the foot segment. Differences between children in the DMD and CP groups were found for the thigh and leg segments. Patients in the DMD group only differ from the healthy group as regards the thigh segment (Table 2). However, Figure 3 shows that the sum of the absolute angles (thigh, leg and foot) does not present statistically significant differences between these groups (*p* > 0.05).

**FIGURE 4:**
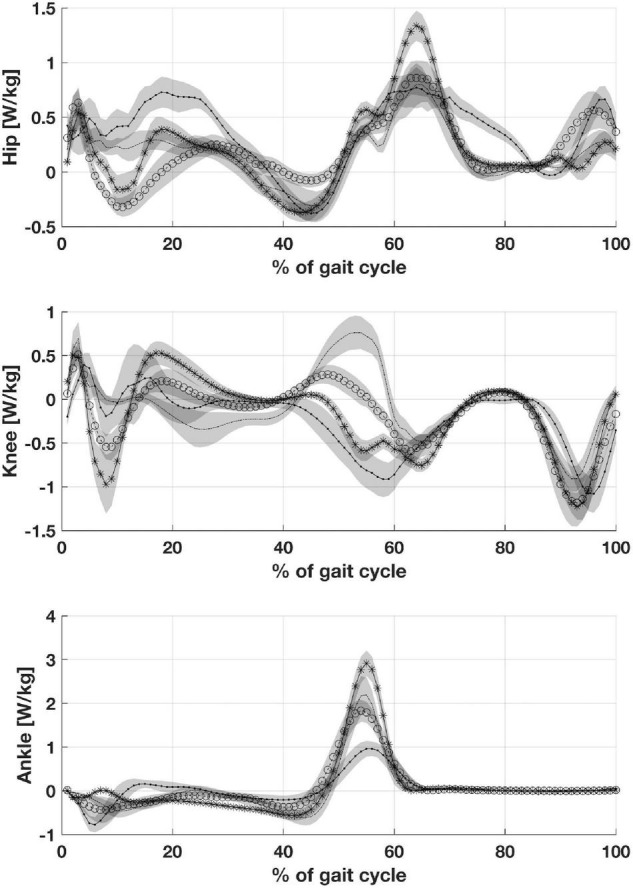
Depicts joints power: Hip joint power, Knee joint power and Ankle joint power. CP, participants with Cerebral Palsy; CMT, participants with Charcot Marie Tooth; DMD, participants with Duchenne Muscular Dystrophy; normal gait (healthy, H).

Patients with CP showed an increase in maximum hip power generation during the stance phase when compared to peers with CMT and DMD. Maximum knee power absorbed during stance was lower in children with CP than in participants with CMT and DMD. The children with CP showed maximum ankle power generation and absorption during the stance phase statistically lower than healthy children (Figure 4).

## Discussion

In the present study, we conducted gait analysis on children affected by three different pathologies (CP, CMT, and DMD), representing paradigmatic examples corresponding to damage mainly involving the upper motoneuron, the lower motoneuron and the muscles, respectively. We analyzed spatial and temporal parameters, kinematics and kinetics of gait in children with CMT, DMD, and CP compared to a control group. The stride velocity of the three pathologies (CMT, DMD and CP) was not statistically significant compared to the healthy group, which allows us to underline how the three groups are comparable from the functional perspective.

We studied kinematics, analyzing lower limb relative and absolute angles with respect to the vertical axis. The study of relative angles evidenced the distinctive solutions peculiar to each pathologic group; at the same time, the study of absolute angles highlighted the crucial role of the pelvis and foot segments with respect to the typical pattern shown by the thigh and the shank.

Furthermore, it is noteworthy that standard gait analysis reports merge absolute segment orientation in space, like the pelvis or the foot progression angle, with relative joint angles of the hip, knee, and ankle. Another interesting aspect is the special functional role of some body regions: the pelvis embodies the relationship between the lower limbs and the upper body (Amori et al., 2015); and the foot relates the body to the environment.

We observed increasing hip flexion with the increase of pelvis anteversion and vice versa. The absolute pelvis orientation in space and the hip angles are connected. At the same time, the knee mediates the attitude of the pelvis and the foot, reducing the range of motion as in the case of reduction of ankle range in conjunction with the pelvis anteversion attitude (Figure 2). Furthermore, the knee angle tended to shift in flexion or extension in relation to pelvis anteversion or retroversion, respectively. The above-mentioned pathological factor restricts the biomechanical relationship to a particular path. It is impossible to distinguish the contributions of two elements: biomechanics on one side and pathological conditions on the other, in both their central and peripheral expressions (Crenna, 1998). Children with CP that are affected mainly by selective motor control deficits and muscle weakness showed a marked reduction of dorsal flexion in stance and increased plantar flexion in pre-swing. This behavior allows storage and release of the mechanical energy in the muscle structure, conserving energy on the vertical plane (Holt et al., 2000; Fonseca et al., 2001). Children with Duchenne dystrophy showed similar ankle behavior. Yet, in that case, even if the mechanism of passive mechanical energy exploitation is similar, the cause is different because it is due to the decline of contractile fiber in muscles and their relative weakness (Romano et al., 2019). In children with Charcot-Marie-Tooth, we observed a reduction of the ankle range of motion, probably linked mainly to both muscular and articular degeneration. In all the cases, the specific anatomical changes represent constraining elements for gait function.

It is possible to explain the differences in pelvis behavior if we consider the previously mentioned factors in mediation with the upper body. The balancing of the pelvis implies a fine-tuning of muscle activities to stabilize the segment on the two spherical hip joints in a dynamic equilibrium compatible with gait progression. Pelvic anteversion in children with CP could be interpreted as a simplification of the pelvis stabilization in stance phase, hanging on the hamstring muscles, reaching maximum anteversion during the monopodic support gait phase. In children with Duchenne, where the main problem is the weakness and not the deficit of fine motor control, the double bump is present, but it is in phase with the control group, a sort of boost of functional activities (Romano et al., 2019). Children with Charcot-Marie-Tooth present a tendency toward pelvic retroversion. It is possible to observe similar attitudes in the gait of children who are blind from birth (Gazzellini et al., 2016). It is an attitude linked with a cautious gait in which the dynamic aspects are restrained. The causes are dissimilar, that is, in blind children, the uncertainty stems from the reduction of exteroception information, while in the children studied here, it is due to the decrease of information from the foot engaging the terrain. What is common to the two situations is the absence of information from a specific sensory channel. We can speculate about the role of another element which may influence these gait differences: perception. The absolute angles of thigh, leg and foot showed a more consistent behavior throughout the different pathologies. Only children with CP differentiated from the other groups during the final swing phase for the hip and during stance for the foot.

It is intriguing to note that if we sum the thigh and the leg orientation with respect to the vertical axis in the three disease groups, any differences between the three pathologies are canceled. We can hypothesize that these time series represent the invariance necessary to achieve an efficient gait, using all the peculiar available resources, both central and peripheral. When all the lower limb segments were considered together, a slight variation during stance induced by the foot orientation differentiates mainly children with CP from children with CMT. The characteristics of the foot condition in these two pathologies resulted from central and peripheral disease, respectively. The nature of these pathologies leads to opposite feet musculoskeletal abnormalities, which influence the strike of the foot on the ground: flat feet for CP and cavus feet in CMT. However, it should be noted that this work has certain limitations relating to the number of patients assessed, even though DMD and CMT diseases can be classed as rare diseases, qualifying this work as an exploratory study. Overall, our study suggests that the pelvis and the foot attitude play a crucial role in determining the biomechanical configuration for all four groups analyzed. Physical orthopaedic and rehabilitative treatment should consider the personal biomechanical configuration in terms of constrains of the gait. Each group showed a particular solution for balancing body segments, exploiting the available residual resources of the organism, both peripheral and central. Meanwhile, the spatial orientation of the thigh and leg were linked together in a sort of biomechanical invariance independent of the studied pathology. Both elements seem to contribute to a body configuration compatible with bipedal gait.

## Data Availability Statement

The raw data supporting the conclusions of this article will be made available by the authors, without undue reservation.

## Ethics Statement

The studies involving human participants were reviewed and approved by “Bambino Gesù” Children’s Hospital. Written informed consent to participate in this study was provided by the participants’ legal guardian/next of kin.

## Author Contributions

SM, MF, AR, AP, SS, EC, and MP managed the overall project (conceptualization, methodology, and interpretation). SM and MP performed the data preprocessing, statistical analysis, results interpretation, and prepared the original manuscript draft. AP, MF, and AR performed the Gait Analysis data acquisition and database maintenance. GV and TS were responsible for the recruitment and clinical examinations and results interpretation. All authors critically reviewed, read, and approved the submitted version of the manuscript.

## Conflict of Interest

The authors declare that the research was conducted in the absence of any commercial or financial relationships that could be construed as a potential conflict of interest.

## Publisher’s Note

All claims expressed in this article are solely those of the authors and do not necessarily represent those of their affiliated organizations, or those of the publisher, the editors and the reviewers. Any product that may be evaluated in this article, or claim that may be made by its manufacturer, is not guaranteed or endorsed by the publisher.
